# Supplementary material for: PEGylated Magnetic Nano-Assemblies as Contrast Agents for Effective T2-Weighted MR Imaging
Source: Nanomaterials (Basel). 2019 Mar 11;9(3):410. doi: 10.3390/nano9030410 (PMC6473972; doi:10.3390/nano9030410)
Supplement: Supplementary file 1 [file nanomaterials-09-00410-s001.pdf]

# Supplementary Nanomaterials: PEGylated magnetic nano-assemblies as contrast agents for effective T2weighted MR imaging

**Byunghoon Kang[1], Jaewoo Lim[2,3], Hye-young Son[4,5], Yuna Choi[4], Taejoon Kang[2,3], Juyeon Jung[2,3], Yong-Min Huh[4,6*], Seungjoo Haam[1*], and Eun-Kyung Lim[2,3,6*]**

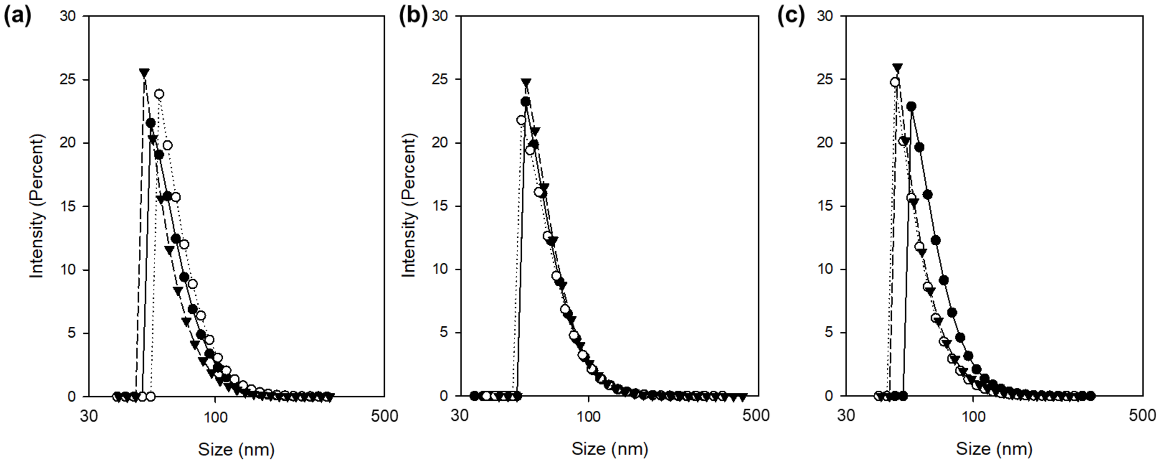


**Figure S1.** The size analysis graphs of PEGylated MNs over 44 days (a): 0 day, b): 30 day and c): 44day). All measurements were repeated three times.
